# KIF11 promotes cell proliferation via ERBB2/PI3K/AKT signaling pathway in gallbladder cancer

**DOI:** 10.7150/ijbs.54074

**Published:** 2021-01-01

**Authors:** Dang Wei, Bian Rui, Fan Qingquan, Cai Chen, Hu Yun ping, Song Xiaoling, Weng Hao, Gu Jun

**Affiliations:** 1 Department of General Surgery and Laboratory of General Surgery, Xinhua Hospital Affiliated to Shanghai Jiao Tong University School of Medicine, 1665 Kongjiang Road, Shanghai 200092, China; 2 Department of Biliary-Pancreatic Surgery, Renji Hospital, School of Medicine, Shanghai Jiao Tong University, 160 Pujian Road, Shanghai, 200127, China.; 3 Shanghai Key Laboratory of Biliary Tract Disease Research, 1665 Kongjiang Road, Shanghai 200092, China; 4 State Key Laboratory of Oncogenes and Related Genes, Shanghai Cancer Institute, School of Medicine, Shanghai Jiao Tong University, Shanghai, 200127, China.; 5 Key Laboratory of Hepatosplenic Surgery, Ministry of Education, Harbin Medical University, Harbin, China

## Abstract

Proliferation is one of the significant hallmarks of gallbladder cancer, which is a relatively rare but fatal malignance. Aim of this study was to examine the biological impact and molecular mechanism of the candidate hub-gene on the proliferation and tumorigenesis of gallbladder cancer. We analyzed the differentially expressed genes and the correlation between these genes with MKI67, and showed that KIF11 is one of the major upregulated regulators of proliferation in gallbladder cancer (GBC). The Gene Ontology, Gene Sets Enrichment Analysis and KEGG Pathway analysis indicated that KIF11 may promote GBC cell proliferation through the ERBB2/PI3K/AKT signaling pathway. Gain-of-function and loss-of-function assay demonstrated that KIF11 regulated GBC cell cycle and cancer cell proliferation in vitro. GBC cells exhibited G2M phase cell cycle arrest, cell proliferation and clone formation ability reduction after treatment with Monastrol, a specific inhibitor of KIF11. Xenograft model showed that KIF11 promotes GBC growth in vivo. Rescue experiments showed that KIF11-induced GBC cell proliferation dependented on ERBB2/PI3K/AKT pathway. Moreover, we found that H3K27ac signals are enriched among the promoter region of KIF11 in the UCSC Genome Browser Database. Differentially expressed analysis showed that EP300, a major histone acetyltransferase modifying H3K27ac signal, is highly expressed in gallbladder cancer and correlation analysis illustrated that EP300 is positively related with KIF11 in almost all the cancer types. We further found that KIF11 was significantly downregulated in a dose-dependent and time-dependent manner after histone acetylation inhibitor treatment. The present results highlight that high KIF11 expression promotes GBC cell proliferation through the ERBB2/PI3K/AKT signaling pathway. The findings may help deepen our understanding of mechanism underlying GBC cancer development and development of novel diagnostic and therapeutic target.

## Introduction

Gallbladder cancer (GBC) is a rare, highly malignant tumor^1^. GBC is perilous partly because it is often diagnosed delayed in its course, when the tumors are already enormous enough to cause blockage and invade nearby structures^2^. Owing to its insensitivity to chemotherapy and radiotherapy, the most effective treatment of this disease is curative resection in early stage^3^. However, only 20% of patients are diagnosed at an early stage, and median survival of advanced GBC is no more than one year^1^, ^4^. Clearly, better early detection methods, and identification of selective therapeutic targets are urgently needed.

Proliferation is one of the significant hallmarks of cancer development and progression^5^. Mitosis requires the microtubule-based cytoskeleton and this cellular physiology is significant not only for GBC but also for amount of other lethal malignancies^6^. Various chemotherapeutic agents, such as Vinca alkaloids, epothilones and taxanes, have been shown to exert their antitumor activity via inhibiting microtubule dynamics^7^. However, these chemotherapy agents are limited in the clinic due to its non-selectivity^8^. This has spurred efforts to find and target microtubule associated proteins (MAPs) whose repression would arrest mitosis without producing toxicity.

In order to identify potential biomarker for the early diagnosis and treatment of gallbladder cancer, the expression data of 19 pairs of GBC tissue and paired normal tissue were collected from the Gene Expression Omnibus (GEO) database^9^, ^10^. Among the upregulated mRNAs, we characterized the KIF11 (Kinesin Family Member 11, also known as EG-5 or kinesin-5) which a key role in the GBC development and progression. KIF11 is a mitotic kinesin that is responsible for forming and maintaining the bipolar spindle^11^. Inhibition of KIF11 function results in the inability of cells to proceed through mitosis and results in cell cycle arrest and subsequent cell death^12^. Besides, KIF11 was shown to be involved in cell movement and transport of secretory proteins from the Golgi complex to the cell surface^13^. Although the role of KIF11 in the malignant behavior of GBC cells has not been fully elucidated, the evidence that it is indispensable for mitotic as well as for cell movement demonstrated that it may play an especially critical role. We therefore aimed to illustrate the functional role and regulatory mechanism of KIF11 in GBC.

## Materials and Methods

### Cohort Datasets, cell lines and agents

RNA-seq dataset GSE139682 and microarray data GSE76633 were download from the Gene Expression Omnibus (GEO) database (http://www.ncbi.nlm.nih.gov/geo). A total of 19 pairs GBC and adjacent normal samples were used for differentially expression analysis.

GBC cell lines NOZ, EH-GB-1, GBC-SD and SGC-996 were purchased from the cell bank of Shanghai Institutes for Biological Sciences, Chinese Academy of Sciences (Shanghai, China). Cells were grown in DMEM medium (Gibco, NY, USA) containing 10% fetal calf serum (Gibco, NY, USA). All cells were routinely cultured at 37 °C, 5% CO2.

The histone acetyltransferase (HAT) inhibitor C646 was purchased from MCE (Medchem Express, USA). The inhibitor was dissolved in DMSO and diluted into four gradients of concentration (5μM, 10μM, 15μM, 20μM). The KIF11 specific inhibitor Monastrol was purchased from MCE (Medchem Express, USA). The inhibitor was also dissolved in DMSO and diluted into four gradients of concentration (5μM, 10μM, 15μM, 20μM).

### Differentially Expressed Gene (DEG) Analysis, Correlation analysis, Gene Ontology (GO), Kyoto Encyclopedia of Genes and Genomes (KEGG) pathway enrichment analysis and Gene Set Enrichment Analysis(GSEA)

The DEseq2 package was applied to DEG analysis based on HTSeq-Counts data from GSE139682^14^. Linear model for microarray (limma) was used for differential gene expression analysis in GSE76633^15^. The DEGs were defined as genes with a log2 fold change > 1 and P-value <0.05. The Venn Diagram package was used for generation of common differentially expressed genes between two data sets. The GEPIA2 database was used to generate the Pearson correlation value between MKI67 and DEGSs^16^. Gene Ontology (GO) and Kyoto Encyclopedia of Genes and Genomes (KEGG) pathway enrichment analysis were conducted by the clusterProfiler package in R to investigate the potential biologic functions of differentially expressed genes^17^. Functional categories with an adjusted P value less than 0.05 were deemed as significant pathways. GSEA analysis was performed with the GSEA software (http://software.broadinstitute.org/gsea/index.jsp)^18^.

### Plasmids, lentivirus particles and regents

Small Interfering RNA (siRNA) were synthesized by Ribobio Company (Guangzhou, China). The KIF11 siRNA sequences were as follows: siRNA#1: 5′-GCACGTCCTGTTACACC-3′, and siRNA#2: 5′-GCACGTCCTGTTACACCAA-3′. siRNA was transfected into cells using RFect Reagent (Changzhou Biogenerating Biotechnologies corporation, China) according to the directions provided by the manufacturers. Full-length cDNA of KIF11 was cloned by Genechem Company (Shanghai, China). The ERBB2-expressing plasmid is a generous gift from Xuechuan Li (Shanghai Key Laboratory of Biliary Tract Disease Research). SiRNA lentivirus of KIF11 gene, which included one target siRNA lentivirus and a negative control siRNA lentivirus, was synthesized by Shanghai Genechem Co., Ltd.

### Cell cycle assay

Cell cycle assay was performed using propidium iodide (PI) staining (Beyotime, Shanghai, China). Cells treated with siRNA or negative control are collected and washed twice in PBS. Then, cells are fixed in 75% ethanol at 4℃ overnight. At last, cells are treated with RNase A, stained with propidium iodide (PI) and analyzed by flow cytometry using MODFIT LT software (Verity Software House).

### CCK-8 assay and colony formation assay

Cell proliferation was assessed with the CCK-8 assay following the manufacturer's instructions. Cells were seeded in 96-well plates at the density of 1000 cells/well, CCK-8 (10 μl) was added to each well, and incubated for 2h. The cell proliferation curves were plotted after assessing absorbance at 450 nm.

500 cells/well were individually seeded into 6-well plates and incubated for 2 weeks to allow colony formation. The culture medium was changed every three days. After that, colonies were fixed with 4% paraformaldehyde and stained with 0.1% crystal violet for 30 min. Then the colonies (with more than 50 cells) were observed under a microscope.

### Xenografted animals

Female nude mice (4-6 weeks) were purchased from the Shanghai SLRC laboratory Animal Co, Ltd. (Shanghai, China). In order to assessment the tumor-forming capacity of the GBC cell, we successively used lentiviral siRNA to transfect cells and Puromycin to select stably transfected cells. Validation and verification of knock-down was performed using Western Blot. Subsequently, 1ⅹ10^6^ NOZ cells (NC, lenti-siRNA-KIF11) were implanted subcutaneously into the left armpit of nude mice (5 mice per group). The widest diameter and the narrowest diameter of xenograft tumor was measured every week by caliper measurements. The tumor volume was calculated by the following formula: tumor volume = 4π / 3 × (width / 2)^2^ × (length / 2). After 4 weeks, tumors were dissected, weighed and photographed. Immunohistochemistry (IHC) analysis was performed in xenograft tumor tissues obtained from mice.

### Real-time quantitative PCR

Total RNA was obtained from tumor cells with TRIzol extraction (Takara, Shiga, Japan) following the manufacturer's directions. Real-time PCR was carried out using SYBR Green PCR kit (Takara, Shiga, Japan) and analyzed with the StepOne Plus system (Applied Biosystems, Foster City, CA). GAPDH was used as an endogenous control. The primer sequence of KIF11 for the forward primer was 5′-TCCCTTGGCTGGTATAATTCCA-3′ and for the reverse primer was 5′-GTTACGGGGATCATCAAACATCT-3'.

### Western blot analysis

The total cellular protein was extracted using RIPA lysis buffer (Beyotime, China). Then, total proteins were separated on a 10% SDS-PAGE gel and transferred electrophoretically to PFDV membrane (Millipore, Bedford, MA, USA) at 100 V for 100 minutes. The blots were blocked in 5% milk for 1 hour, and then incubated with specific primary antibodies at 4°C overnight. Membranes were then incubated with corresponding secondary antibodies for 1 hour and visualized using the enhanced chemiluminescent detection reagent. Western blot analysis was conducted using anti-KIF11, ERBB2, Ki67, PCNA, PI3K, p-AKT, AKT, H3K27ac and GAPDH antibodies. (ERBB2, PI3K, p-AKT, AKT, H3K27ac were purchased from CST, USA. KIF11 was purchased from Proteintech, USA. Others were purchased from Abclonal, USA).

### Statistical analysis

All statistical analyses were performed using R (3.6.0 version). Data from the experiments were expressed as the mean ± standard deviation (SD). The experimental data were statistically evaluated using Student's t test or Mann-Whitney U test. Pearson correlation test was used to analyze the linear correlation between two variables. Differences between groups were considered significant if P values were less than 0.05. The P values are uniformly replaced with the following symbols in all the figures: *p < 0.05; **p < 0.01; ***p < 0.001.

## Results

### 1. KIF11 is expressed at high levels in GBC compared with adjacent normal tissue

**Figure 1:**
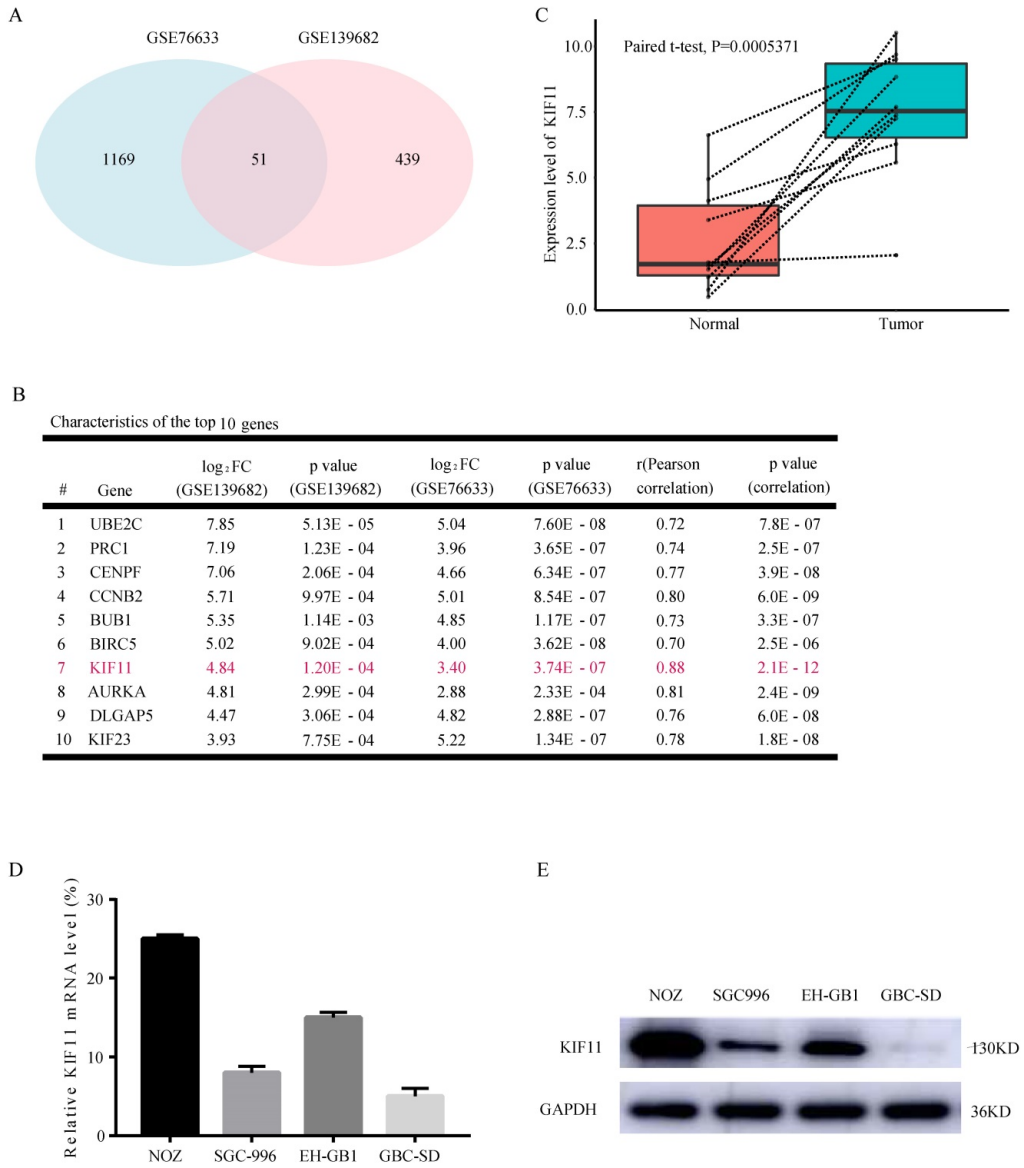
** KIF11 is expressed at high levels in gallbladder cancer compared with adjacent normal tissue.** (A) Veen plot displaying the common differentially expressed genes between GSE76633 and GSE139682. (B) The top 10 upregulated DEGs with correlation values more than 0.7 are listed. (C) The expression level of KIF11 in 10 paired gallbladder cancer sample in GSE139682. (D, E) Endogenous mRNA (D) and protein (E) levels of KIF11 in gallbladder cancer cell lines. DEGs, differentially expressed genes.

Looking for the validated target of GBC, we collected two data sets, GSE76633 and GSE139682 from the GEO database. An analysis of these gene expression profiles (collectively 19 GBC and 19 normal tissues) revealed differential expression for 1220 genes in GSE76633 and 490 genes in GSE139682. As shown in Figure 1A, 51 genes are changed in both data sets. In order to investigate the most important regulator of proliferation in GBC, we analyzed correlations between all the 51 differentially expressed genes with proliferation potential (Ki67). We identified KIF11 as the most significant regulator after filtering out those genes with correlation value less than 0.7 and P value less than 0.05 (Figure 1B). We also performed mRNA-level analysis of KIF11 in 10 paired tumor-normal samples in GSE139682 (Figure 1C). KIF11 mRNA and protein were further quantified by qPCR and western blot in GBC cell lines (Figure 1D and E). The results showed that expression level of KIF11 was relatively high in NOZ and EH-GB1 and relatively low in GBC-SD and SGC-996. Collectively, the consequences above display that KIF11 might be a key driving gene of the GBC development.

### 2. KIF11 promotes cell proliferation by accelerating G2/M cell cycle progression

**Figure 2:**
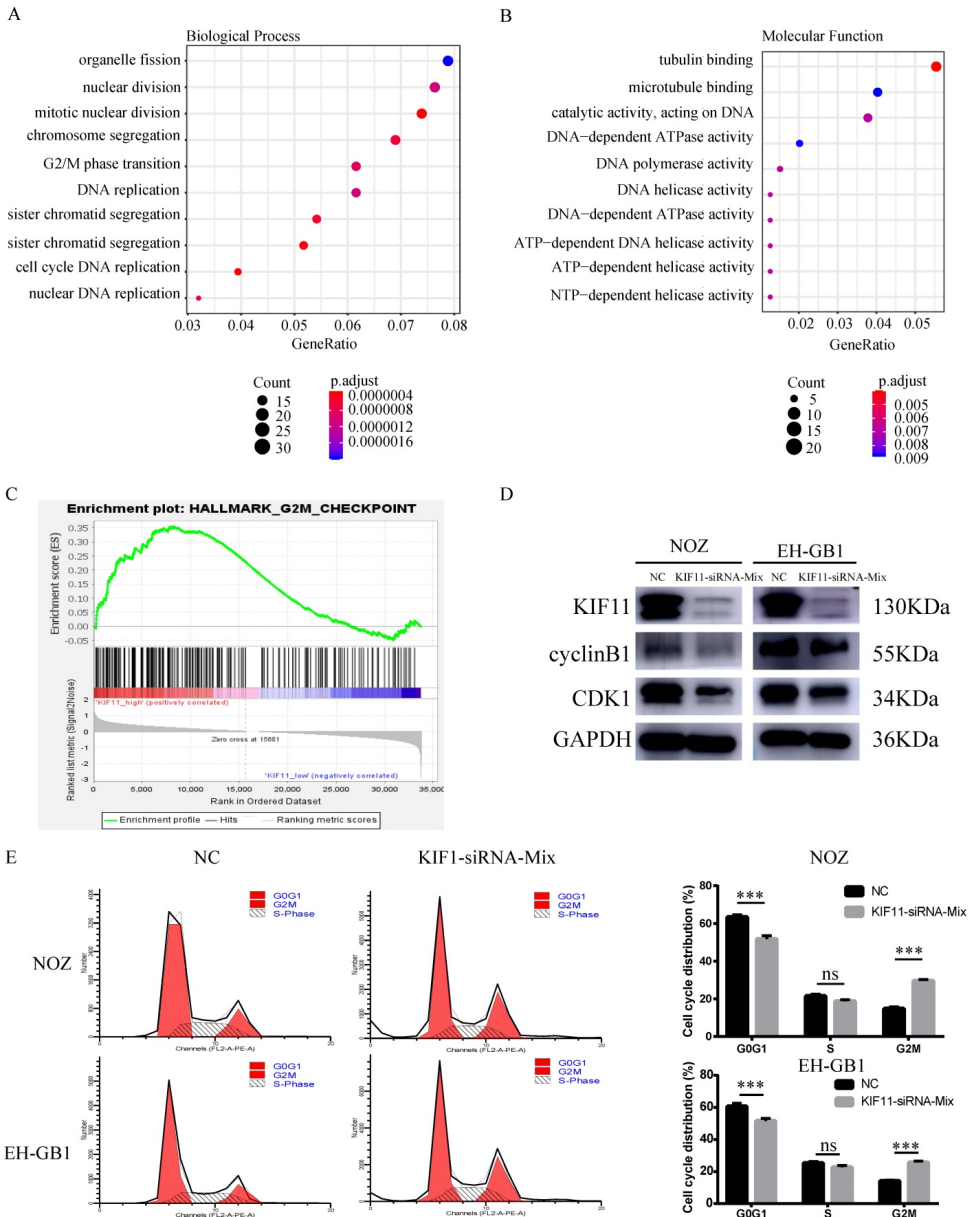
** G2M cell-cycle block induced by KIF11 down-regulation in NOZ and EH-GB1 cells.** (A, B) Gene Ontology (GO) analysis of differentially expressed genes between high KIF11 cancer tissue and low KIF11 cancer tissue. (C) Gene Sets Enrichment Analysis of differentially expressed genes between high KIF11 cancer tissue and low KIF11 cancer tissue using hallmark gene sets. (D) Immunoblotting is shown for cyclinB1 and CDK1. (E) Cell-cycle analysis was performed by flow cytometry.

Ten gallbladder cancer samples were divided into two groups according to the median value of KIF11 mRNA level and then differentially expressed gene analysis was performed. Gene Ontology analysis and Gene Set Enrichment Analysis revealed an enrichment of transcripts related to cell division and mitosis and G2M transition (Figure 2A-C). Therefore, we analyzed the expression of cycle-related protein by western blot. The results showed that protein expression level of Cyclin B1 and CDK1 were downregulated after KIF11 siRNA mixture treatment (Figure 2D). Moreover, the flow cytometry analysis illustrated that the cell cycle was arrested in the G2/M stage in NOZ and EH-GB1 transfected with KIF11 siRNA mixture (Figure 2E).

### 3. KIF11 promotes GBC cells proliferation *in vitro*

Based on the above results, we examined the effect of KIF11 in GBC cells by knocking down the expression of KIF11. To minimize off-target effects of siRNA, we used two pairs of siRNAs, which target different KIF11 mRNA sequences.

**Figure 3:**
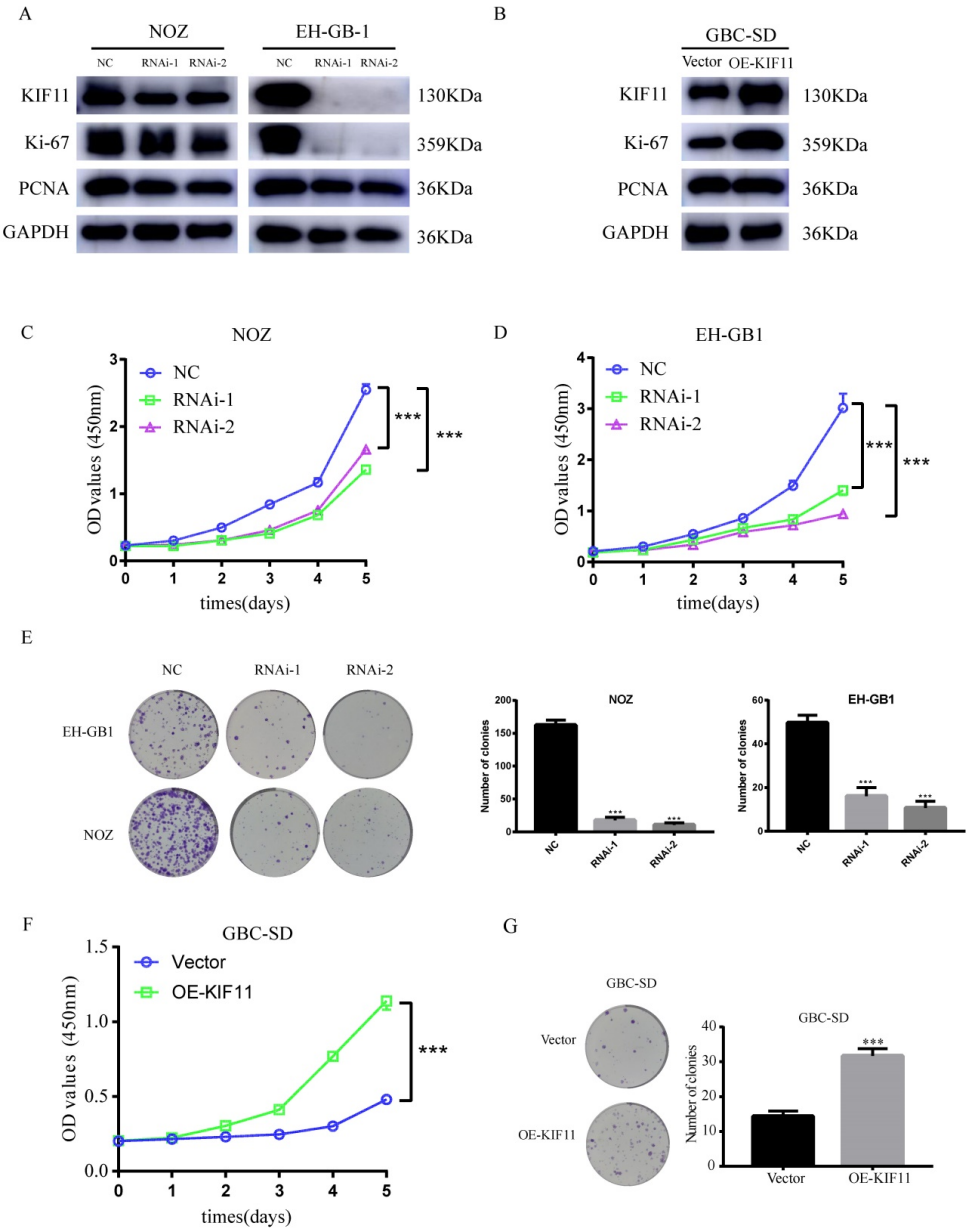
** Effect of the knockdown and overexpression of KIF11 on proliferation of gallbladder cancer cells.** (A, B) Ki-67, PCNA expression are detected by western blot after KIF11 knockdown or overexpression. (C-E) CCK8 assay (C, D) and colony formation assay (E) after KIF11 knockdown in NOZ and EH-GB1 cells. (F, G) CCK8 assay (F) and colony formation assay (G) after KIF11 overexpression in GBC-SD cell.

The knockdown efficiency was validated by western blot. We found that more than 50% reduction of KIF11protein expression in NOZ and EH-GB1 cell lines (Figure 3A). CCK-8 and colony formation assays were utilized to test the functionality of KIF11 in GBC cells. Significant slowdown in proliferation of GBC cells was observed as a result of the KIF11 knockdown (Figure 3C-E). Moreover, we examined the proliferation associated markers, Ki67 and proliferating cell nuclear antigen (PCNA), by western blot and found that knockdown of KIF11 induced downregulation of Ki67 and PCNA in NOZ and EH-GB1 cells (Figure 3A). We also overexpressed KIF11 in GBC-SD cell to complete gain-of-function assays. As shown in Figure 3 F-G, KIF11 overexpression promoted proliferation and upregulated the Ki67 and PCNA protein level in GBC cells. Overall, the results demonstrated that KIF11 could promote GBC cells proliferation *in vitro*.

### 4. Monastrol induced G2M arrest and proliferation inhibition via downregulation of the ERBB2/PI3K/AKT signaling pathway

**Figure 4:**
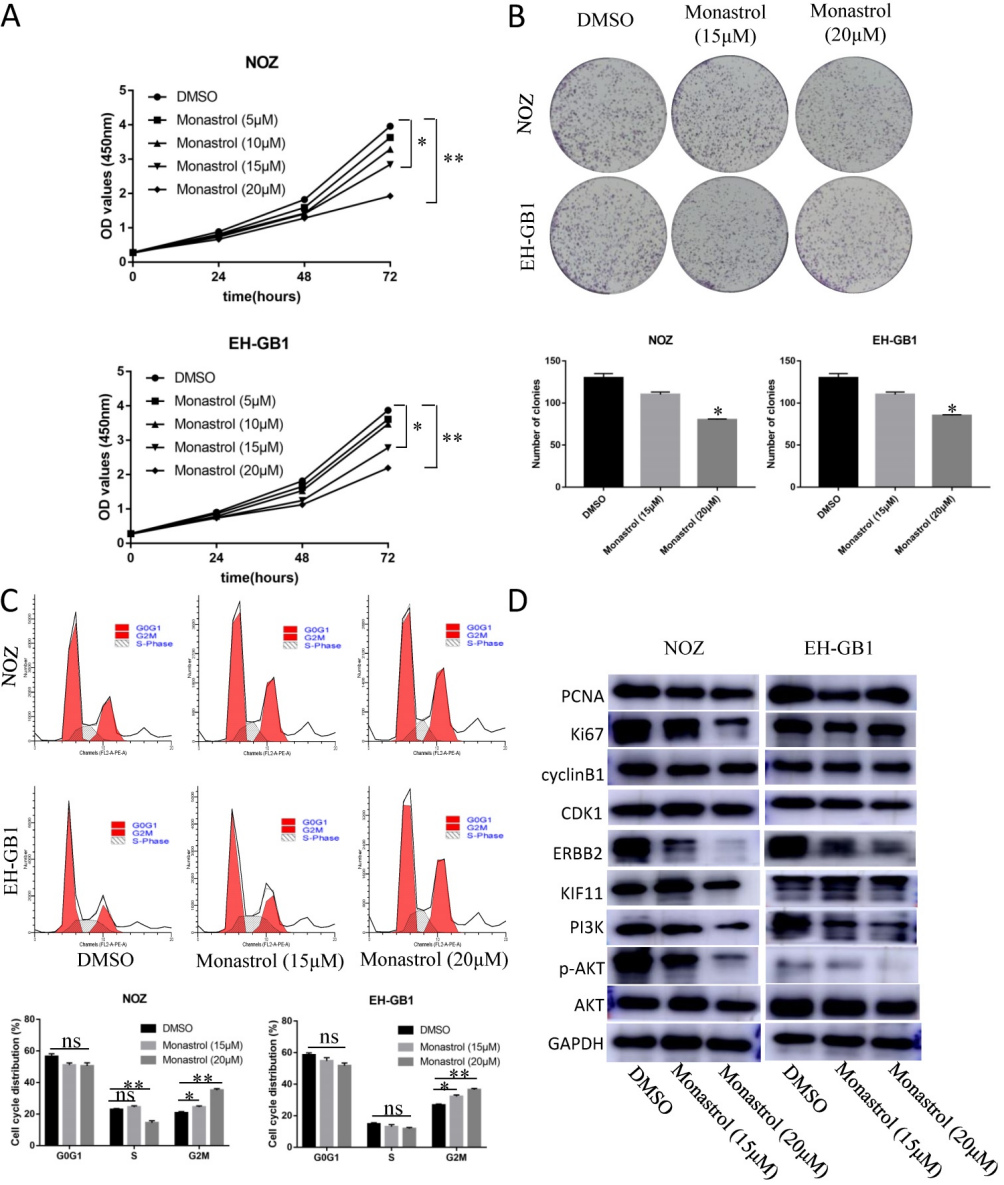
** Monastrol induced G2M arrest and proliferation inhibition via downregulation of the ERBB2/PI3K/AKT signaling pathway.** (A) The cell growth curve of NOZ and EH-GB1 exposed to different concentration Monastrol (0, 5, 10, 15, 20 μM) for different lengths of time (0h, 24h, 48h, 72h). (B) Clonogenic capacity of cells was measured after 48‐h treatment with indicated doses of Monastrol (15μM and 20 μM). (C) Cell cycle distribution after 48 hours of Monastrol treatment (15μM and 20 μM). (D) The immune blots show the proliferation related proteins (Ki67, PCNA), cell cycle related proteins (cyclinB1, CDK1) and the ERBB2/PI3K/AKT signaling pathway proteins levels in GBC cells after treating with Monastrol (15 and 20 μM, 48h).

In order to prove that KIF11 is a druggable target, we purchased KIF11 specific inhibitor Monastrol to assay the effect of this drug on GBC cells' cell cycle and proliferation ability. Monastrol binds to a long loop that is specific to the Eg5 (also known as KIF11 or kinesin-5) kinesin family, and allosterically inhibits ATPase activity of the kinesin^19^. Based on the reference IC50, we set the Monastrol concentration gradient (0, 5, 10, 15, 20 μM) to treat the GBC cells for different lengths of time (0h, 24h, 48h, 72h). CCK8 assay demonstrated that 15μM and 20μM Monastrol can significantly inhibit cell growth in NOZ and EH-GB1 cells (Figure 4A). Then, we select these two effective concentrations to perform plate clone formation assay and cell cycle distribution experiment. After treatment with Monastrol for 48h, the clonogenic capacities of NOZ and EH-GB1 are decreased compared with negative control group (Figure 4B). Flow analysis shows that Monastrol results in G2M phase arrest (Figure 4C). Western Blot illustrated that proliferation related proteins (Ki67, PCNA), cell cycle related proteins (cyclinB1, CDK1) and the ERBB2/PI3K/AKT signaling pathway proteins were reduced by Monastrol treatment (Figure 4D).

### 5. KIF11 facilitates cell proliferation and tumorigenesis *in vivo*

To evaluate the tumor-forming ability of KIF11 *in vivo*, human GBC xenografts were established by subcutaneous injection of approximately 1ⅹ10^6^ KIF11-silenced NOZ cells and negative control cells into the left axillary area of nude mice. Mice models injected with KIF11 knockdown cells exhibited a significant reduction in the growth rate (Figure 4D) as well as the volumes of the xenografted tumors (Figure 4A-C). Immunohistochemical analyses of the human GBC cells xenografted in nude mice showed a low Ki67 and PCNA expression in KIF11-knockdown tumor tissues which were identified with the previous results (Figure 4E).

### 6. KIF11 promotes GBC cells proliferation through activation of ERBB2/PI3K/AKT signaling pathway

**Figure 5:**
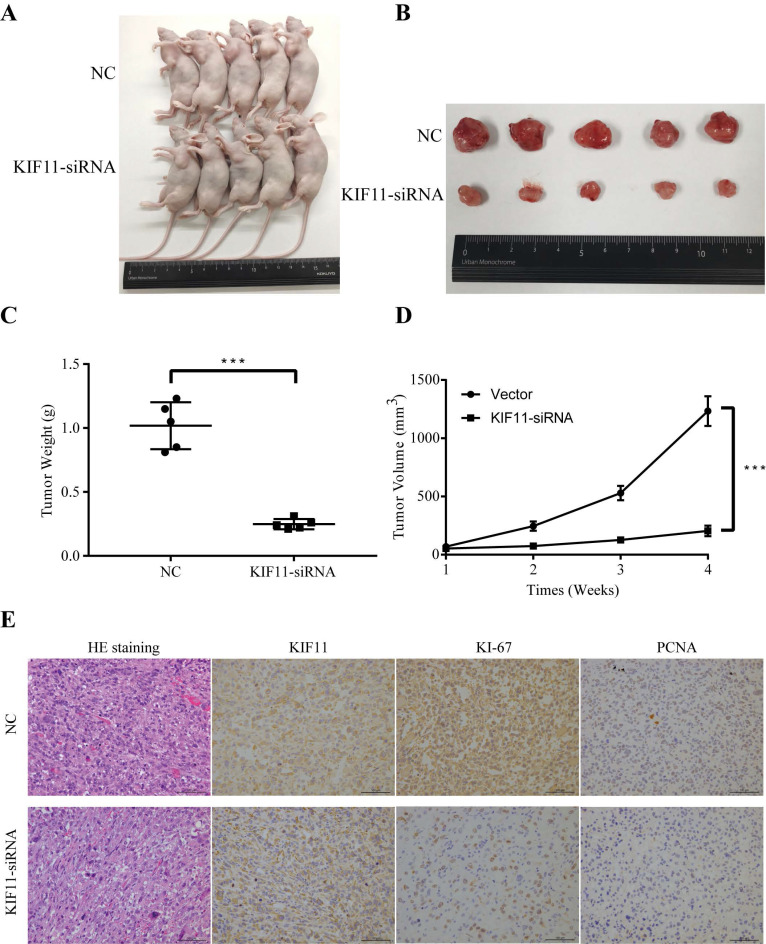
** KIF11 promotes the proliferation of gallbladder cancer xenograft growth in nude mice.** (A) NOZ cells grown as xenograft tumors in nude mice. (B) The morphology of the tumor xenografts. (C) Weight of xenograft tumors harvested from mice. (D) Growth curves of tumor xenografts. (E) Representative images showing HE stain and KIF11, Ki-67, PCNA immunohistochemistry in xenograft tumors.

To explore the downstream signaling pathways regulated by KIF11, differentially expressed genes between high KIF11 expression tumor and low KIF11 expression tumor were then analyzed for enrichment in Kyoto Encyclopedia of Genes and Genomes (KEGG) pathway categories. The result indicated that ERBB2 might be a downstream target of KIF11 (Figure 5A). Furthermore, Gene sets Enrichment Analysis suggests that KIF11-mediated tumor cell proliferation might through the activation of PI3K/AKT signaling pathways (Figure 5B). Previously, Ana Ruiz-Saenz et al. reported that ERBB2 amplification in tumors activates PI3K/AKT signaling pathway^20^. Combined with the results above, we consider that KIF11 might truly be related to ERBB2/PI3K/AKT signaling pathway. Based on the bioinformatic consequences above (Figure 5A-B), we sought to demonstrate whether the promotion proliferation of KIF11 is dependent of ERBB2/PI3K/AKT pathway through biological experiments. As is shown in Figure 5 C-E, cell proliferation ability and colony numbers were reduced in KIF11-knockdown cells, which could be rescued by ERBB2 overexpression. Moreover, the protein levels of KIF11, ERBB2, PI3K, AKT, phosphorylated AKT were detected by western blot in three different samples. We found that KIF11, ERBB2, PI3K, AKT and phosphorylated AKT protein were downregulated after KIF11 siRNA transfection, whereas transfection of ERBB2 plasmids could partially restore this reduction in KIF11 knockdown tumor cells (Figure 5F). In short, the results demonstrated that KIF11 promotes GBC cells proliferation through activation of ERBB2/PI3K/AKT signaling pathway.

### 7. KIF11 is upregulated by histone acetylation modification in GBC cells

**Figure 6:**
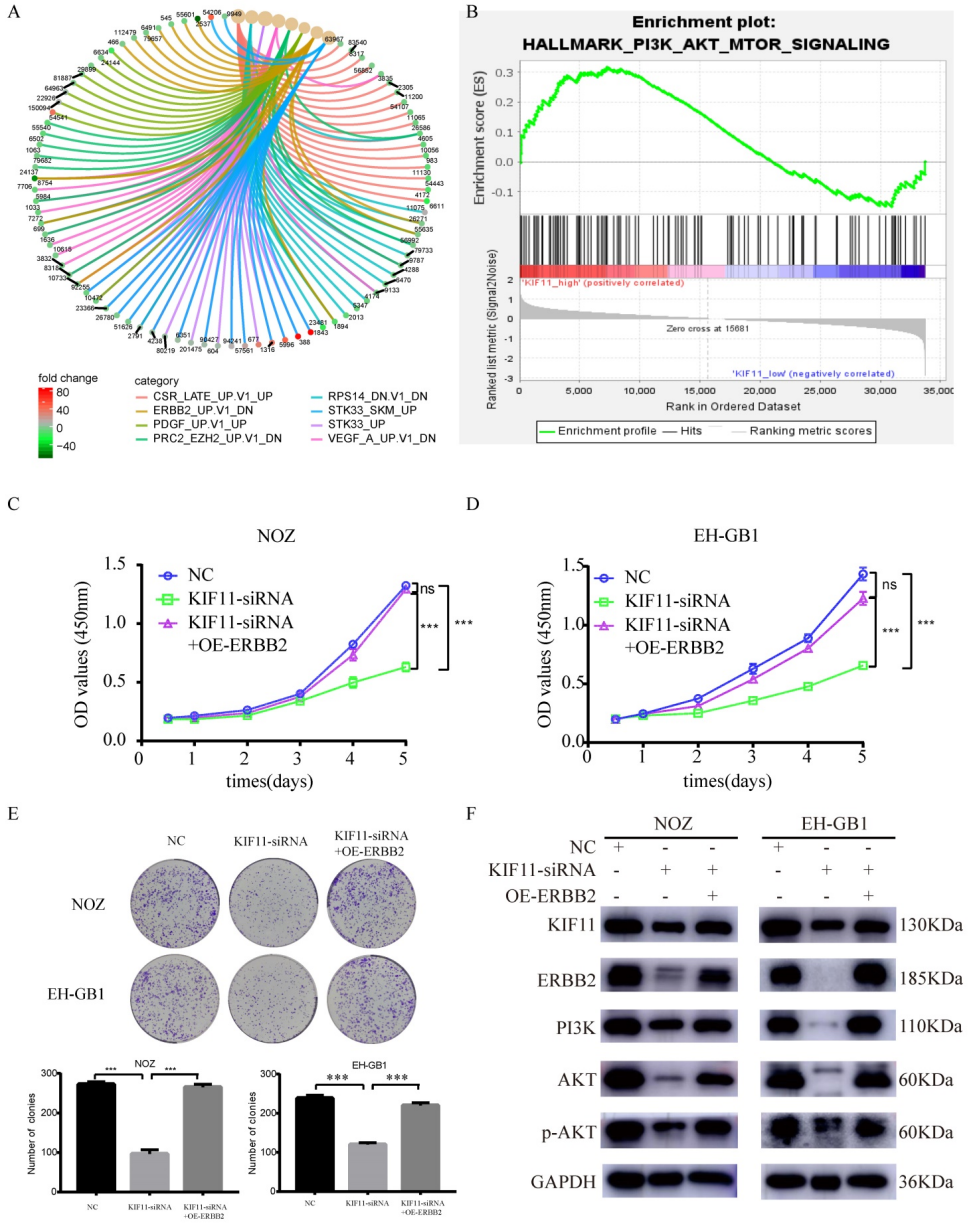
** Proliferation induction by KIF11 is mediated by activation of the ERBB2/PI3K/AKT pathway.** (A) KEGG pathway enrichment analysis of differentially expressed genes between high KIF11 gallbladder cancer tissues and low KIF11 gallbladder cancer tissues. (B) Gene Sets Enrichment Analysis of differentially expressed genes between high KIF11 cancer tissue and low KIF11 cancer tissue using hallmark gene sets. (C-E) CCK8 assay (C, D) and colony formation assay (E) showed that KIF11 knockdown decrease the activity of proliferation while ERBB2 overexpression partially rescued this effect. (F) Western blot analysis of KIF11, ERBB2, PI3K, p-AKT and AKT protein.

Histone acetylation plays a critical role in the regulation of gene transcription, in which acetylation of histones activates gene transcription^21^. To explore the mechanism of high KIF11 expression in GBC, we first analyzed the histone acetylation modification in the promoter of KIF11 by the UCSC genome bioinformatics site (https://genome.ucsc.edu/). H3K27ac enrichment was observed in the promotor region of KIF11, indicating that KIF11 might be regulated by histone acetylation (Figure 6A). It is known that P300 is a major regulator of H3K27ac and C646 is a synthetic small molecule designed to inhibit P300 with high specificity^22^, ^23^. Firstly, we compared the mRNA expression level of EP300 in the gallbladder cancer tissues and adjacent normal tissues. Result showed that EP300 is highly expressed in GBC tissues (Figure S1A). The correlation between EP300 and KIF11 was then analyzed using GEPIA2 database. The correlation analysis revealed significant positive correlations were observed between EP300 and KIF11 in various tumor types (Figure S1B-F). To further confirm above results, we treated the NOZ and EH-GB1 with C646, and the results showed that KIF11 mRNA level was significantly decreased in a dose-dependent and time-dependent manner (Figure 6B-E). Additionally, the results showed that C646 treatment decreased both KIF11 and H3K27ac protein level (Figure 6F), which indicated that KIF11 was regulated by histone acetylation modification.

## Discussion

**Figure 7:**
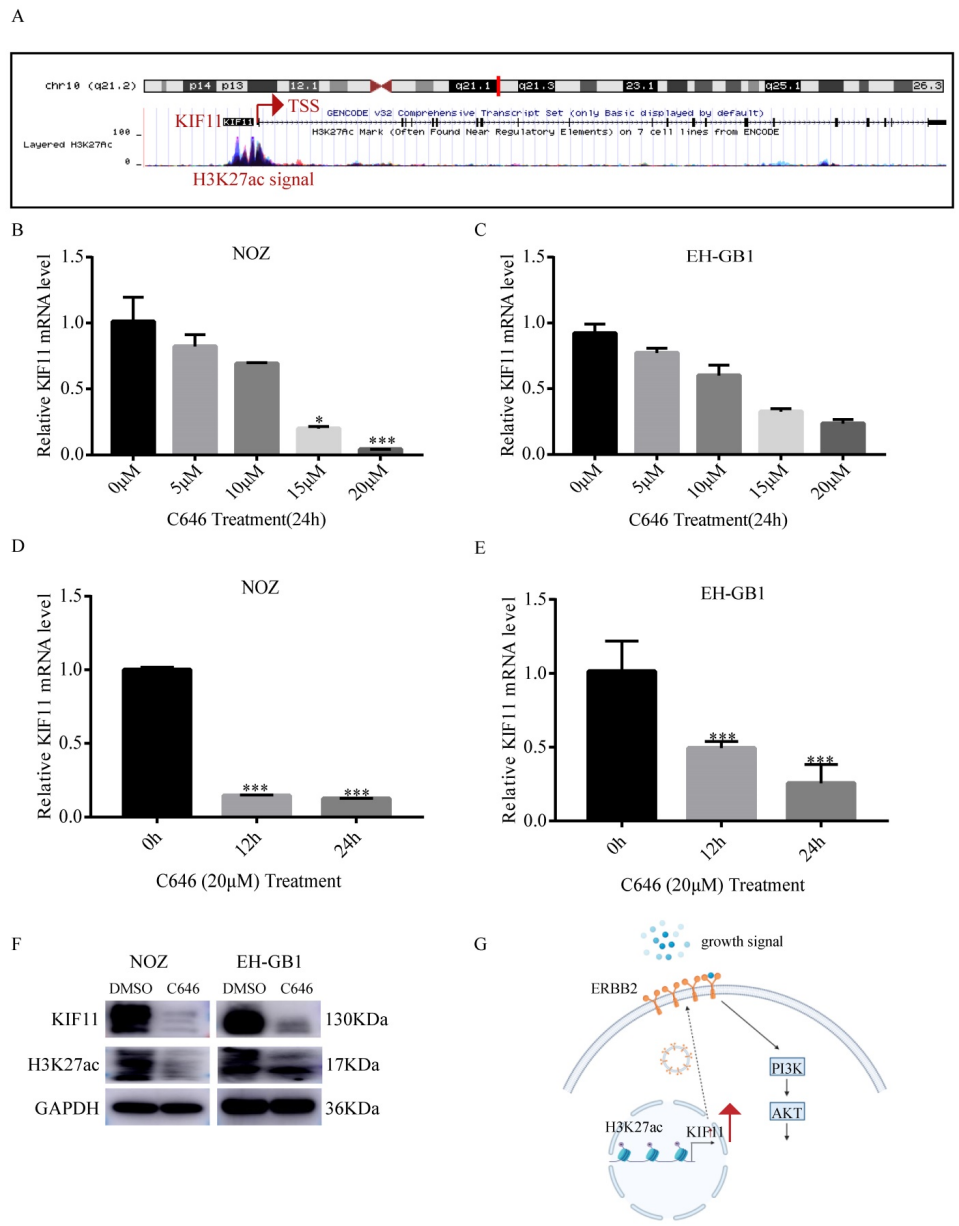
** P300-mediated H3K27ac activates KIF11 expression in gallbladder cancer.** (A) Data from the UCSC genome browser database (https://genome.ucsc.edu/) showed that enrichment of H3K27ac in the promoter of KIF11. (B-C) qRT-PCR analysis of KIF11 mRNA levels in NOZ and EH-GB1 cells treated +/- C646 for 24h with different concentrations. (D,E) The mRNA levels of KIF11 in C646 (20 μM)-treated NOZ (D) and EH-GB1 (E) cells at the indicated time points were measured by qRT-PCR. (F) The KIF11 and H3K27ac protein levels in NOZ and EH-GB1 cells were measured by western blotting after C646 treatment (20μM) for 24 hours. (G) Schematic graph depicting the molecular mechanisms underlying KIF11-mediated pro-proliferation in GBC.

Aim of this paper was to examine the biological impact of the candidate hub-gene on the gallbladder cancer tissues, mainly on the proliferation and tumorigenesis. To pinpoint the most critical gene regulating cell proliferation, we performed differentially expressed genes analysis on the two publicly available RNA-seq and microarray data sets. Pearson correlation analysis further indicated that KIF11 is the most key regulator of proliferation. Quantitative PCR and western blot showed that KIF11 is also upregulated in gallbladder cancer cells. The gain-of-function and loss-of-function approaches were applied to demonstrate that KIF11 promotes cancer cells proliferation in vitro. We also used Monastrol to prove that KIF11 is a druggable target. Moreover, our results from human tumor xenografts in nude mice indicated that inhibition of KIF11 could reduce tumor growth in vivo. We further demonstrated that the biological function of KIF11 was dependent on activation of ERBB2/PI3K/AKT signaling pathway. Based on the above results, we plotted the schematic graph depicting the molecular mechanisms underlying KIF11-mediated pro-proliferation in GBC (Figure 7G).

In this study, we examined for the first time the effects of KIF11 in gallbladder cancer. In accordance with the present results, previous studies have demonstrated that overexpression of KIF11 is related with poor prognosis in patients with clear cell renal cell carcinoma and KIF11 is a driver of proliferation and self-renewal in glioblastoma^24^, ^25^. In addition, Sun et al. reported that KIF11 inhibitor can be a potent chemotherapeutic strategy against gemcitabine resistant gallbladder cancer^26^. Moreover, the present study also constitutes the first description of the association between KIF11 and ERBB2/PI3K/AKT signaling pathway. Li et al. demonstrated that genomic ERBB2/ERBB3 mutations promote immune escape in gallbladder cancer^27^. Albrecht et al. reported that ERBB2 amplification is a potential predictor in gallbladder carcinoma^28^. Corti et al. reported that PI3K/AKT pathway inhibitors proved effective against biliary tract cancers in preclinical studies^29^. The results of our study also support the view that the ERBB2/PI3K/AKT pathway is one of major signaling pathways in gallbladder cancer. Another important finding of this study is that both the transcription and translation of KIF11 are affected by histone acetylation modification. Jin et al. reported that H3K27ac plays crucial roles in the changes of gene expression related breast cancer^30^. Li et al. demonstrated that H3K27ac is elevated in breast cancer^31^. However, there are no current reports of the role of H3K27ac modification in gallbladder cancer. Therefore, our results suggest that H3K27ac might be involved in gallbladder cancer development through regulation of specific gene expression. The other point of interest of the present study is that we combined bioinformatics methods and traditional biological experiments to explore the function of KIF11 in gallbladder cancer. This approach significantly reduced the time and cost of molecule, phenotype and pathway screening compared with previous methods.

Present study has several strengths. First, the findings of this study clearly demonstrated that KIF11 promotes cell proliferation of gallbladder cancer cell through ERBB2/PI3K/AKT signaling pathway. Second, several bioinformatic tools were used to help us to better understanding the biological function and mechanism of KIF11 in gallbladder cancer. Third, we found that histone acetylation modification plays an important role in the development of gallbladder cancer, whereas there exists relatively little research in this field.

The limitations of our study include the following. First, the overall study sample used to perform RNA-seq or microarray was small and thus, the nonspecific DEGs might also be involved into the list. We have taken measures such as literature analysis and extreme value deletion to minimize this error. In addition, we took intersection of the results of differentially expressed genes analysis of two data sets independently to reduce nonspecific DEGs. Second, the corresponding clinical data, especially the survival data are missing in the GSE139682 and GSE76633. This is probably due to the relatively low incidence of gallbladder cancer and poor prognosis. However, it is reassuring that we are collecting follow-up data of patients with gallbladder cancer for filling gaps. Third, the detailed mechanism how KIF11 regulated ERBB2 remained to be discovered. According to Song et al., KIF11 is involved in regulating the transport of β-actin mRNA and cell motility^32^. Wakana et al. reported that KIF11 is important for transport of CARTS from trans-Golgi network to the cell surface^33^. We therefore speculated that KIF11 might enhance the transport of ERBB2 from cytoplasm to cellular membrane. Fourth, we have demonstrated that the H3K27ac modification of the promoter region of KIF11 could increase both the mRNA and protein level of KIF11. However, the specific acetylation sites and enzymes involved in this modification in gallbladder cancer are needed to be elucidated. These interesting directions are beyond the scope of this paper and will be studied in future papers. In spite of its limitations, the study certainly adds to our understanding of the molecular mechanism of gallbladder cancer initiation.

Several major conclusions can be drawn from the findings presented in this study. First, KIF11 is one important driver in gallbladder cancer development. KIF11 likely promotes tumor cell mitosis and cell proliferation through ERBB2/PI3K/AKT signaling pathway. Second, it is well established that KIF11 could be upregulated by histone acetylation. These findings partly explain the rapid growth and poor prognosis of gallbladder cancer. Our findings in concert offer several insights that could be useful for the early detection and novel therapeutic targets development. Continued efforts are needed to make KIF11 more accessible to achieve its clinical application.

## Supplementary Material

Supplementary figures.
