# Supplementary material for: KIF11 promotes cell proliferation via ERBB2/PI3K/AKT signaling pathway in gallbladder cancer
Source: Int J Biol Sci. 2021 Jan 1;17(2):514–26. doi: 10.7150/ijbs.54074 (PMC7893577; doi:10.7150/ijbs.54074)
Supplement: Supplementary file 1 — Supplementary figures. [file ijbsv17p0514s1.pdf]

Figure S1:

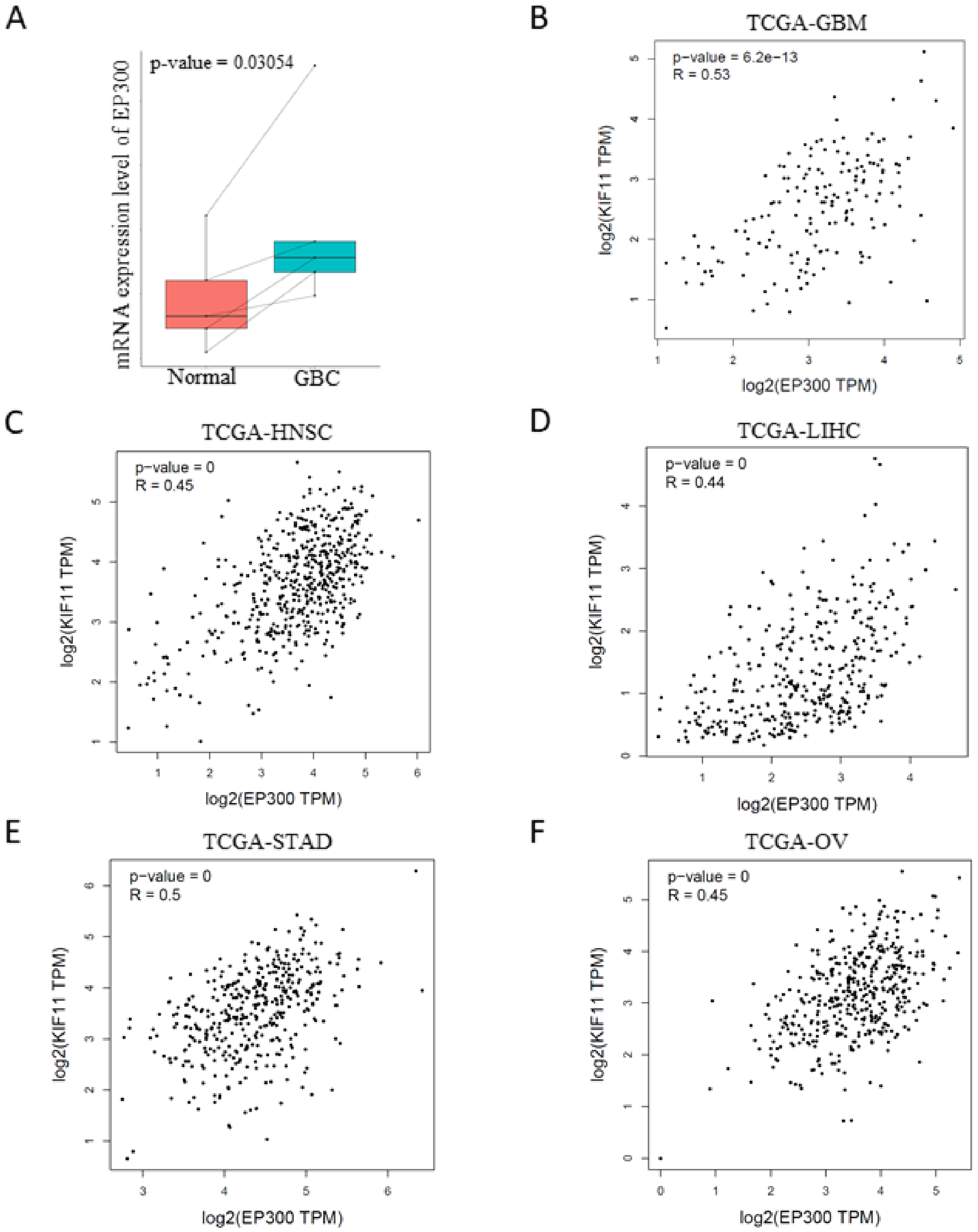

FigureS1:

EP300 is highly expressed and positively related with KIF11 in GBC. (A) The expression level of EP300 in gallbladder cancer and adjacent normal tissues in GSE139682. (B-F) Correlation between the levels of EP300 and KIF11 in five cancer types.
